# Evaluation of gradual adaptation of mixed microalgae consortia cultivation using textile wastewater via fed batch operation

**DOI:** 10.1016/j.btre.2018.e00289

**Published:** 2018-10-27

**Authors:** Gopalakrishnan Kumar, Menghour Huy, Péter Bakonyi, Katalin Bélafi-Bakó, Sang-Hyoun Kim

**Affiliations:** a School of Civil and Environmental Engineering, Yonsei University, Seoul 03722, Republic of Korea; b Institute of Chemistry, Bioscience and Environmental Engineering, Faculty of Science and Technology, University of Stavanger, Box 8600 Forus, 4036 Stavanger, Norway; c Department of Environmental Engineering, Daegu University, Republic of Korea; d Research Institute on Bioengineering, Membrane Technology and Energetics, University of Pannonia, Egyetem ut 10, 8200 Veszprém, Hungary

**Keywords:** Microalgae consortia, Textile wastewater, Decolorization, Harvesting cycle, Growth rate

## Abstract

This study investigated mixed microalgae consortia cultivation in a fed batch reactor using textile wastewater. The results showed 95% of total phosphorus (TP) and 70% of total nitrogen (TN) depletion during the operational period. Algal biomass growth, pollutant removal, and biomass constituents were examined for five cycles of the fed batch operation. The length of the cycles decreased from 30 days to 10 days as the cycle repeated, which implied gradual adaptation of microalgae to textile wastewater. Color of textile wastewater was removed in the range of 68–72% in all the cycles experimented. Microbial algal cultivation with textile wastewater would be a feasible approach for remediation and resource recovery purpose.

## Highlights

• Gradual adaptation of microalgae consortia to textile wastewater was demonstrated.
• Harvesting cycles of microalgae cultivation was examined via fed batch operation.
• Color removal efficiency was achieved in the range of 68–72%.
• Total nitrogen and phosphorous were depleted completely within 7 days of operation.

## 1 Introduction

Energy consumption and demands for the new and efficient technologies that utilize plenty of energy are growing simultaneously. This scenario led to various environmental issues along with pollution urge the environmentalists for the immediate action for the remediation of polluted sites, especially for the wastewater treatment [1]. Wastewater is an immediate and abundant source of plenty of nutrients and macro elements necessary for the cultivation and growth of microalgae. This could be an alternative to reduce the cost of the overall price of the microalgae biofuel [2,3].

Microalgae bioremediation have been proved as an efficient method for the treatment of various kinds of wastewater along with the generation of biomass, which could be subsequently utilized for the production of biofuels and bio products [1,4,5]. Previously, some investigations have been carried out photoautotrophic, heterotrophic and/or mixotrophic cultivation of microalgae biomass with the simultaneous treatment of municipal, brackish, secondary and industrial wastewater as a cheaper nutrient medium (to replace the commercial/expensive bold’s medium) and also renewable approach towards the resource recovery [3,6,7].

In spite of the sustainable tackling of the win-win situation of treatment and biomass production for energy production of microalgae cultivation, prospecting cost-effective, nutrient abundant growth medium is still a big challenge [1,3]. Exploration of textile wastewater (TWW) for the microalgae cultivation is not well studied in the literature, especially in the continuous operation. Recently, the authors showed the feasibility of using TWW in cycle conditions and reported that biomass productivity could reach up to 0.419d^−1^ [3].

Fed batch reactor (FBR) has been successfully adapted for the contaminant and organic wastewater treatment due to the benefits including the easy adaptation, less space occupancy, and relatively low-cost maintenance [7,8]. Revamping FBR technology for the microalgae cultivation has been proposed with the main theme of biomass cultivation in some earlier studies [9,10]. However, not much attention has been paid to the effluent quality regulations of effluent quality (Nitrogen, phosphorous and COD removal) along with the various bio-components (carbohydrate, protein) formation.

Owing to the lack of microalgae studies relevant to the textile wastewater treatment, this investigation studied an efficient operation strategy of the FBR as a sturdy process towards the treatment and also decolorization to be considered as a cost-effective medium for the proficient eco-friendly and environmentally friendly cultivation strategies. Furthermore, limitation in the adaptation of continuous operation of 5 cycles has been performed for the nutrient removal and also biomass accumulation.

## 2 Materials and methods

### 2.1 Isolation and cultivation of mixed microalgae consortia

Mixed microalgae consortia were collected and isolated from Geumho River in Yeongcheon, Republic of Korea. The collected culture was observed under microscope to check the available microalgae. Before used in the real experiment, preliminary cultivation was carried out with Basal Bold’s Medium which was commonly used for freshwater microalgae. The cultivation was conducted inside growth chamber under light cycle of 12 h light/dark cycle with 212.21 ± 22.22 mol.m^−2^.s^-1^ light intensity. Four subcultures were continuously performed resulted in *Chlorella species* was mostly dominated followed by *Scenedesmus species*.

### 2.2 Growth/nutrient medium

Textile wastewater (TWW) was used for the fed batch microalgal growth [3]. No extra nutrients were added during the entire operation. It was obtained from a textile industry located in Daegu, Republic of Korea. Table 1 shows the physical/chemical characteristics.

**Table 1:** Physical/chemical characteristic of textile wastewater used in this study.

Description	Unit	Mean value	Standard deviation
pH		8.7	0.10
Total solid (TS)	g/l	3.11	0.14
Volatile solid (VS)	g/l	2.34	0.21
Total suspended solid (TS)	mg/l	1.8	0.20
Volatile suspended solid (VS)	mg/l	1.61	0.18
Total chemical oxygen demand (T-COD)	g/l	2.2	0.15
Soluble chemical oxygen demand (S-COD)	g/l	1.75	0.12
Total nitrogen (TN)	mg/l	380.5	12
Total phosphorus (TP)	mg/l	94	3

### 2.3 Experimental setup and operating procedures

In fed batch operation, the mixed microalgae consortia were cultivated inside transparent plastic tube which is allowed light to easily penetrate inside photo-bioreactor with a dimension of 14 cm diameter and 43 cm height (39 cm working height). 500 mL of microalgae consortia was mixed with 4000 mL of TWW to make up the final volume (4500 mL working volume) at the start of the experiment. The 3D design of the experimental configuration was shown in Supplementary information. Photo-bioreactors frame was designed with the stainless steel frame of 36 cm length, 30 cm width, and 60 cm height with disposable cardboard covered the whole structure as walls preventing light escaping. The light source was provided with LED lights (model 5630-60SMD cool white, Samsung, South Korea) fixed with a timer and dimer to control the light cycle and light intensity. Light cycle was set as 12 h dark and 12 h light. As well as, the Light intensity was measured at the photo-bioreactor outside the surface area by digital portable light lux meter (UYIGAO, model UA1010B, China) resulted in 170.21 ± 22.22 μmol.m^−2^.s^-1^. Aeration pump (AMAZONPET, model SH-A2, China) was installed with stone sparger at fixed flowrate of 0.2 vvm in order to provide CO_2_ needed for microalgae photosynthesis. As well as, pH was maintained in the range of 8.2–9.0. The study has been carried out in 5 cycles for 95 days (30 days for the 1st cycle, 22 days for the 2nd cycle, 20 days for the 3rd cycle, 13 days for the 4th cycle, and 10 days for the 5th cycle). Each cycle of the experiment was decided when 50% of COD, 95% of TP, and 70% of TN were depleted. After each cycle finish, 2.5 L of the old culture suspension was collected and then 2.5 L of fresh textile wastewater was added for the next cycle to begin with.

### 2.4 Analytical procedures and calculation

Total solds (TS), volatile solids (VS), Total Nitrogen (TN) and Total Phosphorous (TP), total suspended solids (TSS), volatile suspended solids (VSS) and chemical oxygen demands (COD) were analyzed by following Standard Methods [11]. Optical density (OD) for microalgal growth was measured using UV-Spectrophotometer (SHIMADZU, UV-VIS mini 1240, Japan) at 680 and 750 nm.

Total sugar and protein were measured by using the Phenol-sulfuric acid method and Lowry method, respectively [3].

The growth rate was evaluated by Eq. (1):

(1) $$\mu_{0}=\frac{Ln\left({OD}_{f}\right)-Ln({OD}_{i})}{t_{f}-t_{i}}$$

where, OD_i_ = initial optical density at 680 nm, and OD_f_ = final optical density at 680 nm, t_f_ = final time (days), and t_i_ = initial time (days).

Decolorization was evaluated by following Daneshvar et al. (2007). Before checking the color concentration, microalgae sample was filtered using 0.2 μm Watman filter paper in order to separate microalgae from the wastewater. Wavelength detection was run under UV-spectrum mode from 200 to 800 nm wavelength resulted in maximum absorbance (λ_max_) was found at 519 nm. Decolorization efficiency was calculated using Eq. (2):

(2) $$Decolorization\;(\%)=\frac{{ABS}_{i}-{ABS}_{f}}{{ABS}_{i}}\times 100$$

where, ABS_i_ = initial absorbance at 519 nm and ABS_f_ = final absorbance at 519 nm.

CO_2_ fixation was estimated by Eq. (3) [12]:

(3) $$R_{{CO}_{2}}=P∙C_{{CO}_{2}}∙\frac{M_{{CO}_{2}}}{M_{C}}$$

where, $R_{{CO}_{2}}$ is the rate of CO_2_ fixation (g.L^−1^.day^−1^), P is the biomass productivity (g.L^−1^.day^−1^), $M_{{CO}_{2}}$ is the molecular weight of carbon dioxide, $M_{C}$ is the molecular weight of carbon. $C_{{CO}_{2}}$ is the carbon content of microalgae biomass. Microalgae typically consist 50% carbon of the total biomass [13].

The experiment in this study was carried out at least duplicate and results were shown as mean value ± standard deviation. The statistical analyses were conducted using Microsoft Excel 2013 and SigmaPlot 10.0.

## 3 Results and discussions

### 3.1 Growth of the microalgae consortia using TWW

**Fig. 1:**
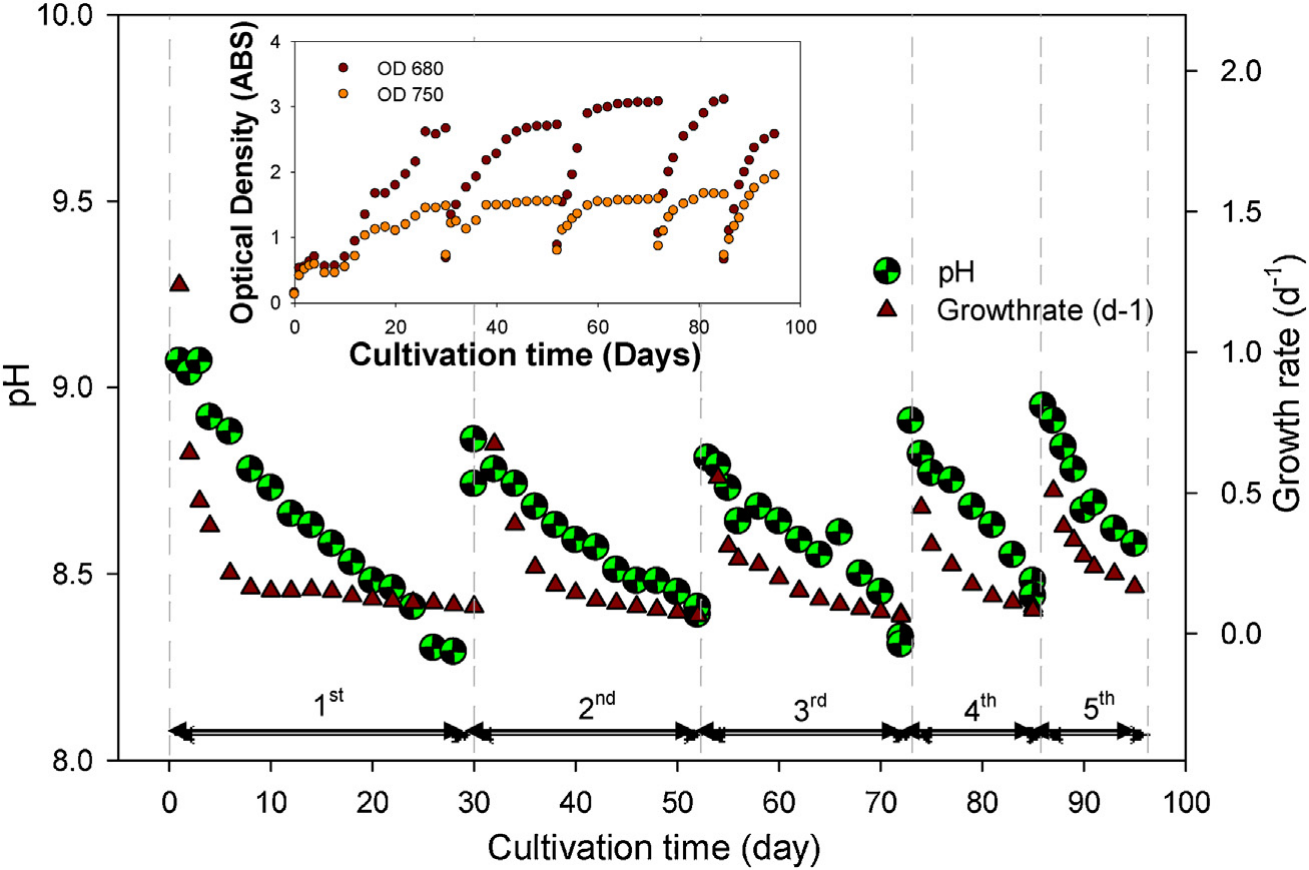
Variation of optical density during 95 days operation of SBR.

Growth estimation by OD is well known to be used in microbiology community since it indicate to measure the suspended biomass inside the liquid sample [14]. The variation of the OD values according to each cycle was shown in Fig. 1. It was illustrated that OD was slightly changed in the first 5 days as lag phase. Log-growth phase started until highest OD value at the 30th day of cultivation of 2.66 and 1.48 for 680 and 750 nm, respectively for the 1st cycle. The 2nd cycle was started with initial optical density of 0.69 and 0.73 and ended with 2.72 and 1.55 for 680 and 750 nm with 22 cultivation days, for the 3rd cycle, dramatically increase of the OD value was observed at the 6 days of cultivation, respectively. This occurred because nutrients of the medium (TWW) are mostly consumed within the log phase period (where the major heterotrophic growth pattern has been observed). TS and VS were obtained at 3.77 g/L and 2.86 g/L. On the 22nd day, OD_680nm_ and OD_750nm_ were raised up 1.083–1.915, respectively.

From the 7th cultivation day, the cultivation was carried on in order to observe the changes and resulted as slightly increased OD value was observed until the 20th day of cultivation as the values of 3.07 and 1.69 were recorded for 680 and 750 nm at the end of the cycle. For the 4th cycle, the cultivation stopped at 13th day (the start of log phase) of cultivation, where 3.11 and 1.65 were noted for OD 680 and 750 nm at the end of the experiment. The 5th cycle was finished in 10 days of cultivation, where 2.57 and 1.95 were marked for OD 680 and 750 nm, respectively. Due to optical density, the peak exponential growth rates were estimated to be 1.24, 0.67, 0.56, 0.45 and 0.51 d^−1^ for the 1st, 2nd, 3rd, 4th and 5th cycle, respectively.

### 3.2 Dynamics of T-N and T-P and organic removal during the FBR operation

**Fig. 2:**
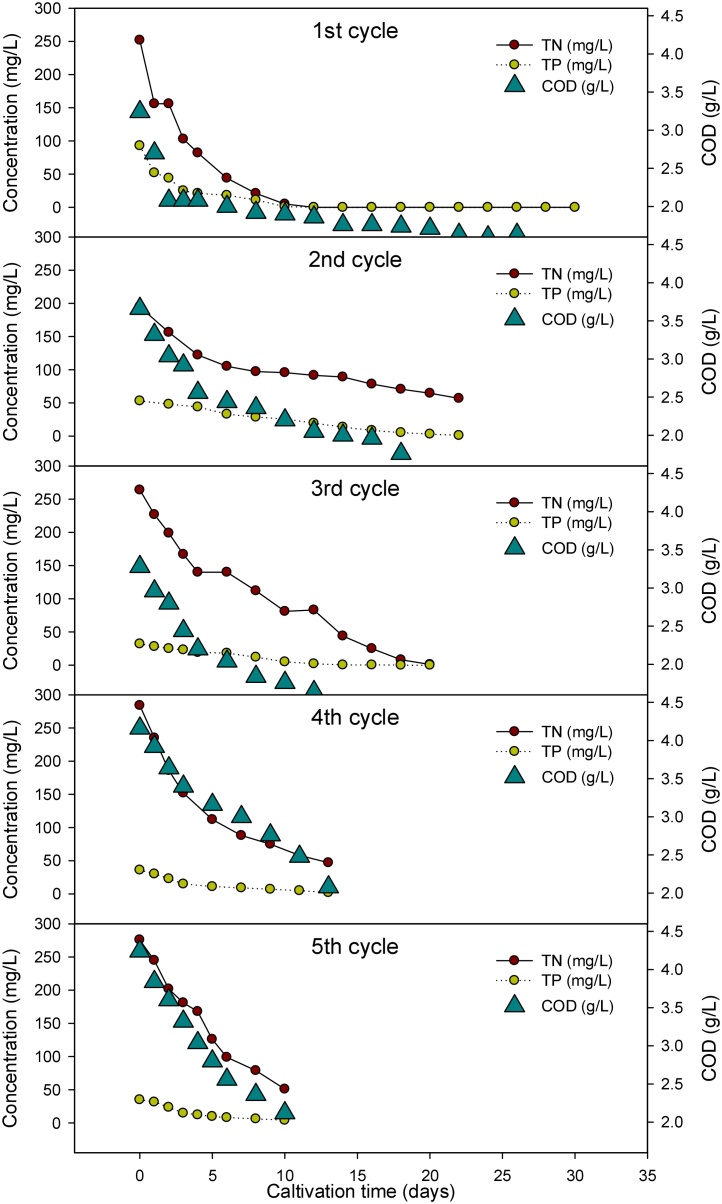
Organic removal for each cycle of FBR operation.

Fig. 2 illustrates the reduction of TN, TP and COD through cultivation time by each cycle. In the 1^st^ cycle, microalgae culture consumed nutrient rapidly in the log phase, where TN and TP were depleted at 12 days of cultivation and COD remained 1.86 g/L. At the end of the 1st cycle, COD remained 1.46 g/L. Due to low concentration of TN (194.8 mg/L) and TP (53 mg/L), TN and TP in the 2nd cycle were dramatically decreased from 95.6 and 25.8 mg/L in 10^th^ day of cultivation. By the end of cycle, 1.76 g/L of COD, 56.8 mg/L of TN and 1 mg/L of TP were only remained inside the growth medium. For the 3rd cycle, rapidly nutrient consumption was observed in the first 8 days of cultivation were 112 and 12 mg/L were remained for TN and TP. Similar results were found as TN and TP were almost depleted after 5th day of the operation [15].

As shown in Table 2. organic removal efficiency was obtained as 52% for COD, 71% for TN and 98% for TP, respectively. TS and VS were noted as 4.16 g/L and 2.97 g/L at the end of the 1st cycle. The 2nd cycle showed the OD_680nm_ and OD_750nm_ were raised up to 3.491 to 1.921 for first 12days. Organic removal efficiency was obtained as 50% for COD, 68.56% for TN and 68.56% for TP. 4.92 g/L and 2.93 g/L were gathered as TS and VS. After 30days of cultivation, the 2nd cycle was conducted. At the end of the cycle at 20th day of cultivation, TP was completely depleted and TN was remained as1 mg/L as well as COD concentration prevailed as 0.96 g/L. At the similar pattern with the previous cycles, microalgae utilized most of the nutrient where it’s reached stationary phase at the 13th day of cultivation. Likewise, 5th cycle started and ended in 10 days, where 2.12 g/L of COD, 51 mg/L of TN and 5 mg/L of TP were noted. Overall organic removal values can be seen in Table 2.

**Table 2:** Organic and nutrient removal and decolorization of each cycle during FBR operation.

Harvesting Cycles	Initial concentration			Final concentration			Removal Efficiency			Decolorization efficiency
	COD	TN	TP	COD	TN	TP	COD	TN	TP	
	g/L	mg/L	mg/L	g/L	mg/L	mg/L	%	%	%	%
**1^st^ (30 days)**	3.24 ± 0.1	252 ± 1.2	93 ± 0.6	1.46 ± 0.1	0	0	54.9 ± 2.0	100	100	68.0
**2^nd^ (22 days)**	3.66 ± 0.1	194.8 ± 2.3	53 ± 1.3	1.76 ± 0.1	56.8 ± 0.9	1 ± 0.3	51.9 ± 1.5	70.8 ± 1.6	98.1 ± 0.6	68.6
**3^rd^ (20 days)**	3.28 ± 0.1	264 ± 2.5	32 ± 0.8	0.96 ± 0.1	1 ± 0.7	0	70.7 ± 0.8	99.6 ± 0.3	100	70.1
**4^th^ (13 days)**	4.16 ± 0.1	284 ± 1.8	36 ± 0.6	2.08 ± 0.2	47 ± 1.2	2 ± 0.5	50 ± 1.4	83.4 ± 1.4	94.4 ± 0.5	71.6
**5^th^ (10 days)**	4.24 ± 0.2	276 ± 1.4	35 ± 0.9	2.12 ± 0.1	51 ± 0.08	4 ± 0.2	50.2 ± 0.9	81.5 ± 0.9	88.5 ± 0.5	72.0

### 3.3 Composition analysis of mixed microalgae consortia on each cycle of FBR operation

The biomass productivity by means of TS, VS, TSS and VSS of each cycle has been evaluated. At the 1st cycle of the experiment, initial TS and VS concentration of microalgae consortia was found to be 1.46 g/L and increased to 3.77 g/L at the end of the cycle of 30 days cultivation. For the 2nd cycle, TS and VS were increased rapidly to 4.14 g/L in 22 days of cultivation. Likewise, TS and VS of 5 and 2.95, 4.84 and 3.99, and 4.85 and 2.37 g/L were noted at the end of each cycle for 3rd, 4th and 5th cycle, respectively.

**Fig. 3:**
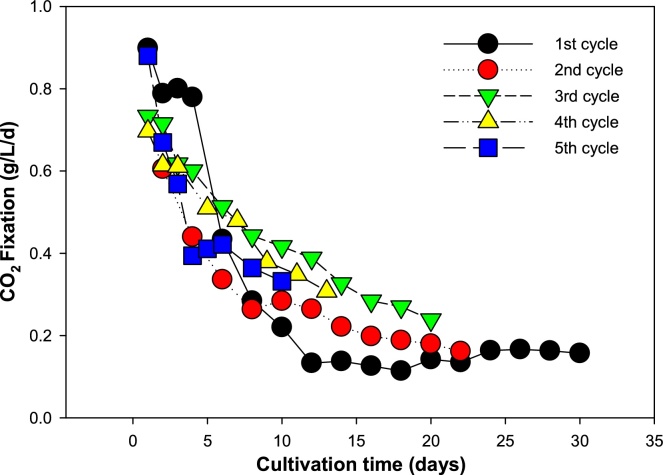
CO_2_ fixation by microalgae for each cycle based on the biomass production.

Protein and carbohydrate accumulated indicated the quality of microalgae grown biomass alongside with remediation as well [19]. The accumulation of protein and carbohydrate are shown in Supplementary information. At the end of the experiment, the accumulated protein and carbohydrate were achieved 2.28 and 3.72, 1.76 and 3.22, 2.63 and 4.57, 1.57 and 4.87, and 1.49 and 4.95 g/L, respectively,

Microalgae have a great potential where it can achieve very high CO_2_ fixation through photosynthesis and give rapid biomass concentrations compared to terrestrial plants [[20], [21], [22]]. As shown in Fig. 3, highest CO_2_ fixation by microalgae was achieved at 0.89, 0.61, 0.73, 0.70 and 0.88 g.L^−1^.d^−1^ with highest biomass productivity of 0.49, 0.33, 0.4, 0.38 and 0.48 g.L^−1^.d^−1^ for 1st to 5th cycle, respectively. Mean value of CO_2_ fixation by microalgae was estimated to be 0.33, 0.38, 0.46, 0.49 and 0.51 g.L^−1^.d^−1^ for 1st to 5th cycle, respectively.

Decolorization was determined at the end of each cycle of the experiment. According to [23], decolorization generally related to the structural modifications of dye molecules in the sample and could be done by adsorption to biomass or biodegradation [24]. Color removal at the 1st to 5th cycle was achieved 68.0, 68.6, 70.1, 71.6 and 72.0%, respectively. The overall result of decolorization could be seen in Table 2.

### 3.4 Prominence of this investigation: discussion

It is one of the critical factors to prospect and select cost-effective growth/nutrient medium towards efficient growth of microalgae for the biofuel/biochemical generation along with the remediation of toxic/nutrient removal such as nitrogen and phosphorous [19]. Over the previous literature survey, Lee et al. [18] reported that using semi-continuous reactor with livestock wastewater as growth medium achieved 0.2 g.L^−1^.d^−1^ of biomass productivity where Yu and Kim et al. (2017) sequencing batch reactor operated achieved 0.315 g.L^−1^.d^−1^ as shown in Table 3. In this study, 0.49 g.L^−1^.d^−1^ of biomass productivity was obtained [7,18]. Increasing biomass concentration inside the reactor would lead to the deprivation of light availability. Thus a balance should be maintained between the generated biomass and the newly growing biomass for the efficient utilization of the nutrient and light towards mass production during the mixotrophic cultivation [25].

**Table 3:** Comparative table with previous studies reported in the literature.

Operation	Substrate	Microalgae species	Biomass productivity (g.L^−1^.d^−1^)	Growth rate (d^−1^)	Removal efficiency			Reference
					COD (%)	TN (%)	TP (%)	
Batch	Institutional wastewater	*Chlorella sp.*	–	0.29	84.86	98.2	70.5	[16]
Batch	Institutional wastewater	*Scenedesmus sp.*	–	0.33	95	99.7	80.5	[16]
Batch	Anaerobic digested dairy manure	*Chlorella sp.*	–	0.41	38.4	75.7	62.5	[17]
Batch	Textile waster	*Mixed microalgae species*	–	0.42	78.78	93.3	100	[3]
Semi-continuous	Livestock wastewater	*Botyococcus braunii*	0.2	–	–	96	85	[18]
Sequencing batch reactor	Livestock wastewater	*Botyococcus braunii*	0.315	0.2	–	83.2	94.1	[7]
Fed batch reactor	Textile wastewater	*Mixed microalgae species*	0.49	1.24	70.7	99.62	100	This study

Some earlier reports mentioned that microalgae concentration at the range of 1.0–1.5 g TS/L cutback nearly 99% of the light to the cell [26]. Thus fed batch operation could avoid such conditions and favor the specific growth rate due to the dilution of the biomass with the addition of fresh feed and also limits the nutrient starving conditions. In this report also authors performed fed batch operation for 5 cycles to ensure the light and nutrient availability to the cells inside the reactor while the biomass concentration reaches nearly 1.0–1.5 g TS/L.

Mixed microalgae consortia consumed most of the T-N from the TWW source within 7–8 days of operation in each cycle while exponential growth occurred, except the adaptation at 1^st^ cycle (more than 10 days). The removal values obtained in this study are quite similar and comparable with the other studies [7,18]. For T-P, it was observed that only 4–5 days were needed to achieve more than 90% of the removal efficiency since the initial concentration is slightly lower than T-N, as approximately 50 mg/L. These values are comparatively higher than the values reported by Yu and Kim, 2017, in that study authors studied only 70 and 0.3 mg/L of T-N and T-P [7] and also reported the deficiency of P throughout their experiment. However, in this report, enough P and N concentrations (250 and 50 mg/L) were provided for the better utilization of micro algal cells. Over the literature review, Wang et al. (2017) achieved 75.7, 62.5 and 38.4% for TN, TP and COD removal efficiency by running under batch condition [17]. With the similar pattern, Ansari et al. [16] studied two different species of microalgae using institutional wastewater achieved 98.2 and 99.7, 70.5 and 80.5, and 84.86 and 95% of TN, TP and COD removal efficiency for *Chlorella* sp. and *Scenedesmus* sp. [16]. Moreover, Huy et al. [3] showed 93.3, 100 and 78.78% for TN, TP and COD removal efficiency for textile wastewater [3].

On the other hand, with the semi-continuous reactor operation by *Botyococcus braunii* with livestock wastewater, Lee et al., [18] reported that they obtained 96 and 85% of TN and TP removal efficiency [18]. In addition, Yu and Kim et al., (2017) operated sequencing batch with the same condition of Lee et al., [18], 83.2 and 94.1% were TN and TP removal efficiency were achieved [7]. According the average on the TN and TP consumption rate per day was shown in Fig. 2, switched from one batch to another led to the augmentation in nutrient consumption rate where 6.27 and 0.26 g.L^−1^.d^−1^ were found for the average TN and TP removal rate at the 2nd batch of the operation, respectively. Further operation, average TN and TP removal rate were to 13.15 and 0.12, 18.23 and 0.12, 22.5 and 0.11 g.L^−1^.d^−1^ for 3rd, 4th and 5th cycles respectively. This attributes to the fact that fed batch mode for cultivation of microalgae is preferable for efficient nutrient removal as well as mass cultivation.

pH will increase during the photosynthesis due to the uptake of CO_2_ in aqueous phase which would be converted to HCO_3_^−^, however, in our study only ambient air has been used as aeration revealed the lower pH values. pH value change during the entire operation of 95 days is depicted in Fig. 1. pH changed during the entire operation was maintained between 8.0–9.0, which is also in accordance with other researchers reported than microalgae was generally cultivated within pH 7–9 in the literature [27].

Textile pollutants are mainly in the form of colorants added during the dying/phosphating process in the industries. Remediation of TWW via biological agents (bacteria, fungi and microalgae) involve mainly follow the mechanisms of bioaccumulation, bio-adsorption, bio-coagulation and bio-conversion as explained by the previous studies [28,29], This can be explained as that algal cell walls contains various functional groups viz SO_4_^−^, PO_4_^−^, amino, carboxyl and proteins, which are the acting as adhesive to the pollutants present in the TWW by anyone of the mechanisms proposed above. However, more detailed mechanism/pathway for the removal (adsorption/degradation) of the pollutants is not well documents and demand more advanced analysis to deepen the knowledge on this aspect [28]. Based on the above mentioned factors, this study provided the novel insights about the fed batch operation of microalgae cultivation using the TWW towards competent cost-effective strategy for the mass generation of biomass which could be subsequently converted to biofuels and value added chemicals via bio refinery aspect.

## 4 Conclusions

Gradual adaptation of microalgae biomass to TWW as nutrient/growth medium has been demonstrated in this research showed that peak growth rate was achieved as 1.24 d^−1^. Continuous cultivation at a periodic interval aided to improve nutrient consumption rate of TN and TP, respectively. FBR operation performed for the resource recovery showed the selection of cost-effective medium and also operational mode is an essential step towards microalgae cultivation.

## Conflict of interest

Authors declare none.
