# Supplementary material for: Evaluation of gradual adaptation of mixed microalgae consortia cultivation using textile wastewater via fed batch operation
Source: Biotechnol Rep (Amst). 2018 Oct 27;20:e00289. doi: 10.1016/j.btre.2018.e00289 (PMC6218807; doi:10.1016/j.btre.2018.e00289)
Supplement: Supplementary file 1 [file mmc1.docx]

Supplementary Figure captions

Figure S1: 3D configuration of photo-bioreactor

Figure S2: Biomass productivities and Accumulation of Protein and Carbohydrate during 95 days operation of SBR during the FBR operation (TS; total solids, VS: volatile solids, TSS: total suspended solids, VSS: volatile suspended solids, A. Protein: accumulation of Protein, A. Carbohydrate: accumulation of Carbohydrate)


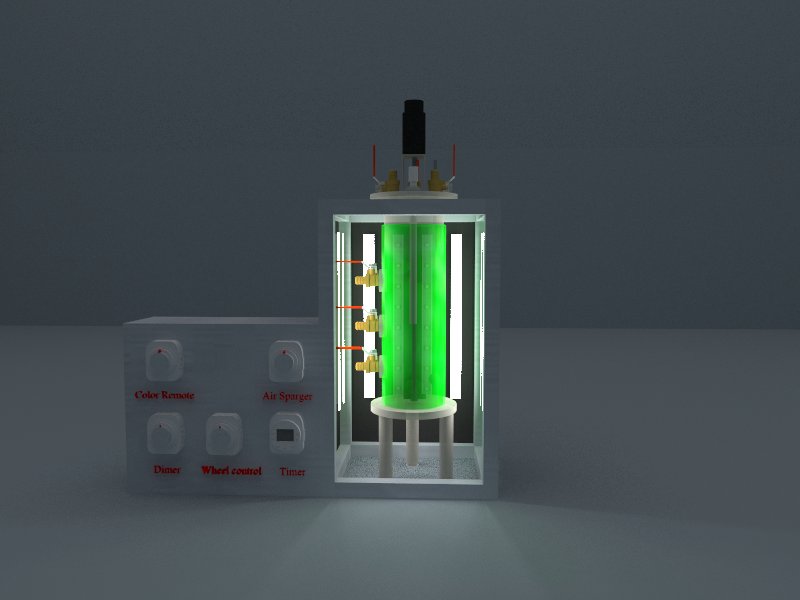


**Fig. S1**

**Fig. S2**
